# A Successful Mode of Preventing Gangrene in Compound Fractures

**Published:** 1802-04

**Authors:** John Crowther

**Affiliations:** resident at Halifax


### *A successful Mode of preventing Gangrene in compound Fractures;*

*As practised by Messrs.* WILLIAM, ROBERT, and JOHN CROWTHER, *in the West Riding of Yorkshire.*

*Communicated by Mr.* JOHN CROWTHER, *resident at Halifax.*

IT may be proper to premife this account of the manner of treating compound fractures, by obferving, that the method now now recommended has been practifed, with the greateft fuccefs, by our anceftors, in Eland, near Halifax, time immemorial, being handed down from father to fon.

When we are called to a patient who has fuffered a compound fracture, (of the leg for example) we firft endeavour, as expeditioufly as poffible, to clear the wound of all extraneous bodies, and then put the limb by a gentle extenfion into a good pofition, the knee being kept at the fame time moderately bent, to favour the relaxation of the mufcles of the fractured limb. While this is performing, we order a large quantity of black bafilicon ointment, made with tar inftead of pitch, to be liquefied in an iron or earthen pan, and made hot, then put into it with all difpatch a large pledget of tow, fufficient if poffible to cover the whole wound, fo as totally to exclude all external air. If one pledget be not fufficient to cover all the lacerated parts, then another muft be applied. Our manner is generally to fpread out the tow, take hold of each end, dip the middle part into the hot ointment, then lift it out, and apply it directly to the wounded part. The leg being raifed and kept in a proper pofition, as faid above, a roller about two inches broad is applied in fuch a manner as to give liberty to the future fwelling of the limb. The roller, before its application, is always moiftened in alegar or vinegar (we generally ufe the former), with a folution of faccharum faturni in it, in the proportion of about half an ounce to a pint of the liquid. We begin at the foot, and roll upwards as high as, or above, the knee, rather tight at the ankle, if the fracture be not there, but always gently wherever the fracture is, taking care at the fame time that the great toe is in a line with the patella, and the fole of the foot nearly fquare with the heel; the latter rule is intended to prevent a retraction of the heel, which would hinder the patient after the cure from ftepping fairly and even upon the ground, when he at firft attempts to walk. Upon this roller we apply pafteboard, cut broad at the top and narrow at the bottom, of the length of the leg, and three or four in number, as occafion may require. Thefe are to be foaked well in warm water, and faftened clofe to the limb by another roller (not applied very tight) of the fame breadth as the former, and moiftened in the like manner. The pafteboard is moiftened that it may, when dry, take the fhape of the limb, and be more eafily and expeditioufly applied at the next dreffing. Then come the fplints, one of which is to be large, and always put underneath the leg, fo as to reach down from the bend of the knee to below the heel, to keep the inferior fractured parts from finking, and thereby prevent a prominence of the fuperior parts of the bones. The fplints are always lined with foft linen, tow, &c. to fill up up all the cavities of the limb, and lie eafy; and this lining is faftened to the fplints, that they may always come off together, and thus expedite the dreffing. And particular care is always taken that a large foft comprefs or pad be placed underneath the heel, left it fhould heat and inflame, and become intolerably painful. And, laftly, we place the limb upon a pillow, and tie it up with three or four pieces of tape at equal diftances, faftened with bow knots, to be the more eafily loofened. If the patient be tolerably eafy, we do not take off the dreffings for one, two, or fometimes three days; but if otherwife, we infpect the wound the following day, to examine the caufe and remove it. The fecond dreffing is made in the fame manner as the firft, with this difference, that the ointment is not applied quite fo hot, nor the bandage fo loofe as before, unlefs inflammation, or other circumftance, fhould indicate the propriety of a contrary treatment. At the third dreffing, the ointment is applied ftill cooler, and fo on; but always continued as a warm dreffing, until there be a free and laudable purulent difcharge; which being once accomplifhed, the ointment is ufed quite cool to the end of the cure, unlefs fome contra-indication fhould arife, fuch as proud flefh, &c. The fame moift bandages, and their manner of application, are generally continued until the inflammation of the limb abates, and then they are ufed in a dry ftate. In about a week or ten days we commonly begin to be more circumfpect at every dreffing in fhaping the limb, by making greater extenfions, bringing the fractured parts of the bones more into contact, clofing the lips of the wound, &c. for, before this time, we are under no great apprehenfion of any little irregularity in the coaptation of the ends of the bones. Indeed, for fome time after this period, we compare the callus to foft glue, which we can fhape and mould nearly as we pleafe; and for this reafon we do not difturb the fracture too much by extenfions, &c. at the firft, while the parts are in an inflamed and irritable ftate. The perfect formation of the callus, and union of the bones of the fractured leg of an adult, of a middle age, we generally find to take place in about twenty-five days, provided there is no lofs of bone. And what is remarkable, we have fometimes known, in fimple fractures, the callus to ftiffen and unite very fuddenly; for inftance, the limb has been fomewhat flexible at the fractured part the preceding day, and ftiff the next.

We always examine the pulfe at every vifit, and form a particular judgment by it; if it be full and ftrong, and not very quick, as from 70 to 90 in a minute, we augur well of the patient; but if it be quick and weak we efteem it a bad fign. In the beginning we prefer coftivenefs to an open body, and and wifh it to remain fo for the firft week or ten days, as we think that it keeps up the patient's ftrength, and produces a vigorous circulation in the veffels and lacerated parts, which it is our endeavour to promote; and has alfo this further advantage, that the limb is in no danger, in the early ftage of the cure, of being difturbed and fhaken by the preparation neceffary for an alvine difcharge. In compound fractures there is ufually a great lofs of blood; but if this fhould not be the cafe, and there come on much fwelling and inflammation, with a full ftrong pulfe, (which in our practice rarely happens) we do not hefitate to bleed freely, and give faline cathartics, fuch as fal. Glauber, &c. and, if neceffary, apply leeches to the tumefied parts, and fomentations, and cataplafms of oatmeal and grounds of beer boiled together, but always the ointment before mentioned upon the wounded parts. We recommend it to our patients to fit up in bed every day, conceiving it to be a great means of both promoting digeftion and preferving the ftrength, and far preferable to their being confined in a fupine pofture for fuch a length of time. The patients are generally kept to their ufual diet, but we forbid beer, wine, and all fpirituous liquors for drink, unlefs they have been much accuftomed to any of them, and then allow a moderate quantity. For common beverage we prefer tea, barley-water, and milk. But if the wounded perfon be of a cold phlegmatic conftitution, and the pulfe weak, we give Peruvian bark liberally, and allow a more generous diet and fome wine, with the intention of ftrengthening the habit, and keeping up the circulation in the wounded and lacerated parts; for we fear nothing more than a pale or fallow complexion, a weak quick pulfe, and defect of a glowing febrile warmth.

As we live in a part of the country which abounds with coal mines and ftone quarries, and is full of manufacturers, many of whom are employed in the direction of dangerous machines worked by fteam-engines, we are frequently called upon to attend very ferious accidents. From the 28th of April, 1798, to the 5th of October, 1800, not fewer than ninety-eight fractures have fallen to my fhare, twenty-eight of which were fractures complicated with a wound; but which were never followed by any gangrenous appearances. The fame fuccefs has attended the practice of my two brothers, William and Robert, for many years.

